# Reduced risk tolerance and cortical excitability following COVID‐19 infection

**DOI:** 10.1111/cns.14879

**Published:** 2024-08-06

**Authors:** Yujing Wang, Haoran Yang, Chongzhi Wang, Ti‐Fei Yuan, Song Zhang

**Affiliations:** ^1^ Department of Psychiatry Tongji Hospital, Tongji University School of Medicine Shanghai China; ^2^ School of Educational Science Chongqing Normal University Chongqing China; ^3^ Shanghai Key Laboratory of Psychotic Disorders Brain Health Institute, National Center for Mental Disorders, Shanghai Mental Health Center, Shanghai Jiao Tong University School of Medicine Shanghai China; ^4^ Department of Anesthesiology Renji Hospital, Shanghai Jiao Tong University School of Medicine Shanghai China

**Keywords:** cortical function, COVID‐19, risk decision‐making, TMS

To the Editor,

The past pandemic of COVID‐19 resulted in long‐lasting changes in human society, both psychologically (e.g., in daily decision‐making)^1^, ^2^ and physiologically.^3^, ^4^ Several neurological and psychiatric complications have been identified in infected individuals and may persist for several months after initial infection.^5^ Here we investigate the potential effects of COVID‐19 infection on risk decision‐making and cortical function in humans.

In our study, we analyzed the records of 26 subjects with a history of COVID‐19 infection and 26 healthy individuals without any exposure to COVID‐19. All of these subjects underwent a choice under risk and ambiguity (CRA) task combined with the transcranial magnetic stimulation (TMS) test to detect the risk decision‐making process and cortical function, respectively. We found that patients who recovered from COVID‐19 showed reduced risk tolerance and hypo‐excitability within the primary motor cortex (M1) compared to healthy controls (HCs). In addition, we also found that risk tolerance and cortical silent period (CSP) were negatively correlated in the infected group.

## MATERIALS AND METHODS

### Participants and study design

The medical records of 26 individuals (≥18 years of age) with a previous infection history of COVID‐19 and 26 age‐matched HCs who received CRA and TMS tests between January 2023 and March 2023 in our department were retrospectively analyzed. There were no differences in age, level of education, sex, alcohol use, nicotine use, sleep quality, emotion, and impulsivity between the two groups (Table 1).

**TABLE 1:** Demographic information and scale scores between infected and uninfected groups.

Characteristics	Participants		Statistic	*p*
	Uninfected (*n* = 31)	Infected (*n* = 31)		
Age, M (IQR), yr	26.37 (22.26,33.28)	30.04 (24.41,34.68)	*Z* = −1.232	0.218
Education, M (IQR), years	16.00 (14.00,20.00)	16.00 (15.00,16.00)	*Z* = −1.748	0.081
Male, No (%)	14 (0.45)	13 (0.42)		1.000
AUDIT, M (IQR)	0.00 (0.00,11.00)	0.00 (0.00,9.00)	*Z* = −0.992	0.321
FTND, M (IQR)	0.00 (0.00,0.00)	0.00 (0.00,0.00)	*Z* = 0.071	0.071
PSQI, M (IQR)	5.00 (3.00,7.00)	5.00 (4.00,7.00)	*Z* = −0.348	0.728
BDI, M (IQR)	8.00 (3.00,13.00)	8.00 (4.00,17.00)	*Z* = −0.564	0.573
BAI, M (IQR)	2.00 (0.00,8.00)	4.00 (1.00,9.00)	*Z* = −1.538	0.124
BIS total, M (IQR)	37.50 (28.33,45.00)	35.00 (20.00,45.00)	*Z* = −0.606	0.165
No planning impulsivity, M (IQR)	37.50 (25.00,52.50)	35.00 (20.00,52.50)	*Z* = −1.389	0.223
Motor impulsivity, M (IQR)	32.50 (22.00.5,40)	32.50 (30.00,42.50)	*Z* = −1.220	0.378
Attention impulsivity, M (IQR)	37.50 (27.50,50.00)	36.67 (26.67,44.17)	*Z* = 0.378	0.545

Abbreviations: AUDIT, alcohol use disorders identification test; BAI, Beck Anxiety Inventory; BDI, Beck Depression Inventory; BIS, Barratt Impulsiveness Scale; FTND, Fagerstrom test of nicotine; IQR, interquartile range; PSQI, Pittsburgh Sleep Quality Index.

This study followed the Strengthening the Reporting of Observational Studies in Epidemiology (STROBE) guidelines. It was approved by the ethics committee of Renji Hospital in Shanghai with a waiver of the need for written informed consent.

### CRA task

In the CRA task, subjects were asked to choose between options that determined the amount (¥35) and the lottery ticket according to their preference. The choice was made in two scenarios: one in which the probability of risk is known, where the lottery directly tells the probability of getting the corresponding amount (¥35, ¥56, ¥140, ¥350, and ¥875), and the probability of not getting the amount (12%, 25%, 38%, 50%, 75%); and a context in which the risk probability is ambiguous, where the probability of getting the corresponding amount and the probability of not getting it are both unclear and equally ambiguous, with 24%, 50%, and 74%, respectively. We used a Linear Subjective Value Model to fit the subjective value of each option of participants under known risk and ambiguity task to derive individual attitudes toward risk and ambiguity,^6^ where *α* = 1 for risk neutrality, *α* > 1 for risk‐seeking, and *α* < 1 for risk aversion; when *α* < 1, a large value of the parameter indicates a lower level of risk aversion and therefore a higher tolerance for risk^7^; *β* = 0 for ambiguity neutrality, *β* > 0 for ambiguity aversion, and *β* < 0 for ambiguity seeking; and *γ* is the inverse temperature reflecting the randomness of participants' choice behavior.

### TMS and electromyography recording

Single‐pulse TMS was performed in the left M1 area with Neuro‐MS (Neurosoft Llc, Ivanovo, Russia) connected to a figure‐of‐eight 100 mm coil. The center of the stimulation coil is placed at LM1, tangential to the scalp, with the handle directed posteriorly and at a 45° angle to the sagittal plane. We then moved the coil around the presumed LM1 area until the relaxed right‐hand abductor pollicis brevis (APB) activity reached the maximum peak‐to‐peak motor evoked potentials (MEPs) amplitudes at the same intensity. This site was determined as the optimal stimulation site.

### TMS procedures

Single‐pulse protocols were used to measure the rest motor threshold (RMT) and CSP. The minimum stimulation intensity that produced an MEP of approximately 50 μV for 5 out of 10 stimuli at the optimal stimulation site was referred to as the RMT. In this study, a suprathreshold intensity of 130% RMT was used. Simultaneously, the subject's APB maintained a 20% maximum voluntary contraction for isometric contraction (assessed and monitored with visual electromyography for feedback). The CSP duration is defined as the time between the onset of MEP evoked by suprathreshold stimulation and the recovery of EMG signal.^8^, ^9^ Each three stimuli were grouped into one session (5 s interval), and a total of three sessions were completed, with CSP duration defined as the average of the nine‐stimulus data.

## RESULTS

**FIGURE 1:**
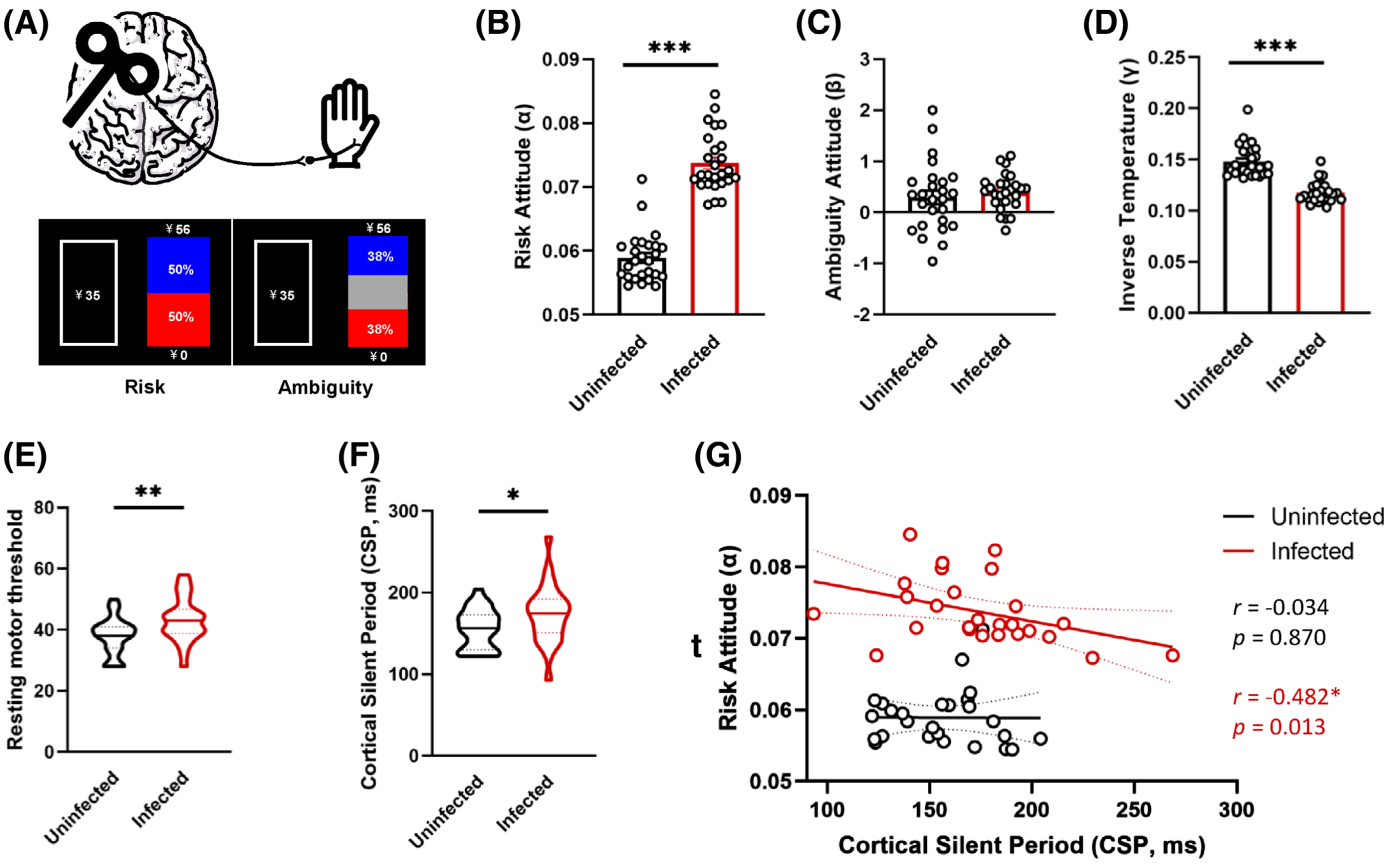
Altered risk decision‐making and cortical plasticity following COVID‐19. (A) TMS and CRA task paradigms. (B–D) Group data show the calculated risk attitude parameter, the ambiguity attitude parameter, and the third subject‐specific parameters (*γ*). (E and F) Quantitative data of the RMT and CSP parameters. (G) There was a negative correlation between risk tolerance and CSP duration in the infected group. CRA, choice under risk and ambiguity; CSP, cortical silent period; RMT, rest motor threshold; TMS, transcranial magnetic stimulation. *p < 0.05, **p < 0.01, ***p < 0.001. Error bars represent SEM.

Compared to HCs, patients recovering from COVID‐19 presented higher risk attitudes (*α*, *Z* = −6.039, *p* < 0.001), but no significant difference was detected in ambiguity risk attitudes (*β*, *Z* = −1.025, *p* = 0.305). The inverse temperature in the COVID‐19 group was significantly lower (*γ*, *Z* = −5.710, *p* < 0.001). Meanwhile, TMS assessment showed higher RMT (*Z* = −2.562, *p* = 0.010) and longer CSP (*Z* = −2.132, *p* = 0.033) in patients after COVID‐19 than those in HCs. In addition, risk attitude and CSP were negatively correlated in the infected group (*r* = −0.482, *p* = 0.013) (Figure 1).

## DISCUSSION

The COVID‐19 pandemic has triggered severe health problems and socioeconomic burdens worldwide. When facing such huge uncertainty, it is of great importance to make optimal risk decisions in daily life. In this situation, we found that the risk tolerance of people with a history of COVID‐19 infection has increased, indicating that they may be more likely to make risk decisions than healthy people, especially when faced with known risks.

Behavioral abnormalities are often accompanied by changes in cortical plasticity. The rMT indicates motor cortex excitability,^10^ and the CSP has been used as an indirect indictor of GABA_B_ receptor‐mediated inhibitory neurotransmission.^11^, ^12^ Moreover, the altered cortical rMT and CSP have been reported in several cognitive disorders.^13^, ^14^ In this cohort, we found a higher rMT and longer CSP of M1 in individuals who recovered from COVID‐19. This could be due to the activation of GABA_B_ interneurons synapsing on pyramidal neurons.

In addition, we found that the CSP was negatively correlated with risk tolerance in the COVID‐19‐infected group, suggesting that the cortical excitability and GABA_B_ neuron function play more vital roles in the risk decision‐making process after COVID‐19 infection. However, larger sample sizes and more neuroimaging studies are needed to explore the possible role of cortical GABA_B_ neurons at different decision‐making stages.

In conclusion, this study improves our understanding of altered risk decision‐making during the COVID‐19 pandemic. The accompanied hypo‐excitability and increased GABAergic inhibition within the cortex provide insights into the mechanisms underlying behavioral disturbances after COVID‐19 and may provide biomarkers to guide rehabilitation.

### TRIAL REGISTRY

The study was registered at https://ClinicalTrials.gov (NCT05961618).

## AUTHOR CONTRIBUTIONS

SZ and T‐FY: Conceptualization, Supervision, Funding acquisition, and Writing – review and editing; SZ, YW, CW, and HY: Project administration; YW, CW, and HY: Data curation and Writing – original draft.

## CONFLICT OF INTEREST STATEMENT

All authors have read and approved the final version of the manuscript. There were no competing interests involved in this study.

## Data Availability

The data that support the findings of this study are available from the corresponding author, Song Zhang, upon reasonable request.
