# Correction to “Reduced Risk Tolerance and Cortical Excitability Following COVID‐19 Infection”

**DOI:** 10.1111/cns.70080

**Published:** 2024-10-11

Y. Wang, H. Yang, C. Wang, T. F. Yuan, and S. Zhang, “Reduced Risk Tolerance and Cortical Excitability Following COVID‐19 Infection,” CNS Neuroscience & Therapeutics 30, no. 8 (2024): e14879.

In the Table 1, *n* = 31 in both uninfected and infected group was incorrect. This should have read: *n* = 26 in uninfected group and *n* = 26 in infected group. Following is the revised table.

**TABLE 1:** Demographic information and scale scores between infected and uninfected groups.

Characteristics	Participants		Statistic	*p*
	Uninfected (*n* = 26)	Infected (*n* = 26)		
Age, M (SD), year	26.96 (5.59)	29.52 (5.27)	*Z* = −1.647	0.100
Education, M (SD), year	16.23 (3.01)	16.04 (1.95)	*Z* = −0.180	0.857
Male, no (%)	13 (0.50)	10 (0.38)	—	0.577
AUDIT, M (SD)	4.04 (6.53)	2.73 (5.56)	*Z* = −0.821	0.412
FTND, M (SD)	0.15 (0.78)	0.81 (2.17)	*Z* = 0.086	0.086
PSQI, M (SD)	5.27 (2.69)	5.73 (2.57)	*Z* = −0.470	0.638
BDI, M (SD)	10.31 (8.46)	10.81 (9.92)	*Z* = −0.055	0.956
BAI, M (SD)	5.69 (9.29)	7.38 (9.90)	*Z* = −1.099	0.272
BIS total, M (SD)	37.76 (16.12)	33.94 (14.59)	*Z* = −0.668	0.504
Noplanning impulsivity, M (SD)	41.73 (24.55)	32.31 (18.32)	*Z* = −1.284	0.199
Motor impulsivity, M (SD)	31.63 (16.88)	35.38 (18.50)	*Z* = −0.926	0.355
Attention impulsivity, M (SD)	39.90 (22.27)	34.13 (11.22)	*Z* = −0.605	0.545

Abbreviations: AUDIT, alcohol use disorders identification test; BAI, Beck Anxiety Inventory; BDI, Beck Depression Inventory; BIS, Barratt Impulsiveness Scale; FTND, Fagerstrom test of nicotine; PSQI, Pittsburgh Sleep Quality Index.

We apologize for this error.

